# Therapy of nodal Follicular Lymphoma (WHO grade 1/2) in clinical stage I/II using response adapted Involved Site Radiotherapy in combination with Obinutuzumab (Gazyvaro) - GAZAI Trial (GAZyvaro and response adapted Involved-site Radiotherapy): a study protocol for a single-arm, non-randomized, open, national, multi-center phase II trial

**DOI:** 10.1186/s13063-019-3614-y

**Published:** 2019-08-30

**Authors:** Laila König, Martin Dreyling, Jan Dürig, Marianne Engelhard, Karin Hohloch, Andreas Viardot, Mathias Witzens-Harig, Meinhard Kieser, Wolfram Klapper, Christiane Pott, Klaus Herfarth

**Affiliations:** 1 Department of Radiation Oncology, University Hospital Heidelberg, Im Neuenheimer Feld 400, 69120 Heidelberg, Germany; 2 Heidelberg Institute of Radiation Oncology (HIRO), Im Neuenheimer Feld 280, Heidelberg, 69120 Germany; 3 Department of Medicine III, University Hospital, Ludwig-Maximilians-University, Munich, Germany; 4 Department of Hematology, University of Essen, Essen, Germany; 5 Department of Radiotherapy, University Hospital of Essen, Essen, Germany; 6 Department of Hematology and Oncology, Kantonspital Graubünden, CH-7000 Chur, Switzerland; 7 Department of Hematology and Oncology, Georg August University, Göttingen, Germany; 8 Department of Internal Medicine III, University Hospital Ulm, Ulm, Germany; 9 Department of Hematology, Oncology and Rheumatology, University Hospital Heidelberg, Heidelberg, Germany; 10 Institute of Medical Biometry and Informatics, University of Heidelberg, Heidelberg, Germany; 11 Department of Pathology, Hematopathology Section and Lymph Node Registry, University of Kiel and University Medical Center Schleswig-Holstein, Kiel, Germany; 12 Department of Medicine 2, University Hospital Schleswig-Holstein, Kiel, Germany

**Keywords:** Follicular lymphoma, Low-dose radiotherapy, Obinutuzumab, Anti-CD20 antibody, Involved site radiotherapy, PET/CT

## Abstract

**Background:**

Large field irradiation had been standard for early-stage follicular lymphoma (FL) for a long time. Although involved field radiotherapy (IF-RT) was recently favored because of the toxicity of large field irradiation, smaller irradiation fields have been accompanied with an increased risk of out-of-field recurrence. The MIR (MabThera^®^ and Involved field Radiation) trial has shown that the combination of IF-RT at a dose of 30–40 Gy with the anti-CD20 antibody rituximab has led to similar efficacy compared with large field irradiation but with markedly reduced side effects. Immune modulating radiation therapy alone using low-dose radiotherapy (LDRT) of 2 × 2 Gy has been shown to be effective in FL. The GAZAI (GAZyvaro and response Adapted Involved-site Radiotherapy) trial aims to prove the efficacy of LDRT in combination with a novel anti-CD20 therapy.

**Methods/design:**

The GAZAI trial is a non-randomized, open, non-controlled, German, multi-center phase II trial that includes patients with early-stage (I and II) nodular FL (grades 1 and 2) confirmed by central histological review. A maximum of 93 patients will be included in the trial. Patients will receive a combined approach of immunotherapy with the fully humanized anti-CD20 antibody obinutuzumab (Gazyvaro^®^) and involved site radiotherapy (IS-RT) with 2 × 2 Gy. The primary endpoint of the trial is the rate of metabolic complete response (CR), based on fludeoxyglucose positron emission tomography/computed tomography, after obinutuzumab and 2 × 2 Gy IS-RT in week 18. Secondary endpoints are morphologic CR rate in weeks 7 and 18 and month 6, progression-free survival, toxicity, recurrence patterns, overall survival, and quality of life. Additionally, minimal residual disease response is assessed. The risk for a potentially higher recurrence rate after LDRT will be minimized by additional salvage radiation up to the “full dose” of 40 Gy for patients who have less than a metabolic CR and morphologic partial response/CR, which will be evaluated in week 18, offering a response-adapted approach.

**Discussion:**

The goal of this trial is a further reduction of the radiation dose in patients with nodal early-stage FL showing a good response to a combination of LDRT and anti-CD20 immunotherapy and a comparison with the currently published MIR trial.

**Trial registration:**

EudraCT number: 2016-002059-89. ClinicalTrials.gov identifier: NCT03341520.

**Electronic supplementary material:**

The online version of this article (10.1186/s13063-019-3614-y) contains supplementary material, which is available to authorized users.

## Background

### Radiation dose

Radiation therapy (RT) alone has been standard for patients with follicular lymphoma (FL) in the early stages. Long-lasting remissions and the potential for cure were the main arguments supporting this approach. In regard to the applied radiation dose, different concepts are discussed internationally. In a prospective British trial published in 2011, the dose effect was evaluated in a randomized fashion in 361 patients with indolent lymphoma [1]. Patients received either 20 × 2 Gy or 12 × 2 Gy involved field RT (IF-RT) without revealing any difference in the local or systemic control. Despite several critical issues in the study design (e.g., heterogeneous histology with 40% non-follicular grade 1/2, previous treatments, and non-standardized follow-up examinations), 24 Gy was then recommended as a standard dose by the European Society for Medical Oncology [2].

Interestingly, immune modulating low-dose radiotherapy (LDRT) alone has shown long-lasting effects in patients with low-grade lymphomas.

Although most patients (73%) in the first retrospective study had advanced disease (stages III and IV), treatment with 2 × 2 Gy yielded an overall response rate (ORR) of 89% (complete response (CR) in 37% and partial response (PR) in 52%) [3]. Subsequently, more patients with low-grade lymphoma received the 2 × 2 Gy scheme: Table 1 [3–8] shows a summary of different trials using 2 × 2 Gy LDRT, and CR rates range from 37% to 82%. In a subset of patients (low tumor burden, fewer previous chemotherapies, age of less than 65 years, and treatment with curative intent), LDRT showed excellent results; ORR and CR rate ranged from 93% to 81% and from 57% to 90%, respectively [5, 6, 8]. The mode of action of 2 × 2 Gy is not completely understood. An induction of the p53 pathway is discussed [9, 10]. Based on a small sample size and a mixed population of patients with FL, *in vivo* imaging led to the speculation that LDRT neutralizes anti-apoptotic effects of the characteristic bcl-2 overexpression in FL cells [11].

**Table 1:** Response rates after 2 × 2 Gy involved field low-dose radiotherapy

Publication	Number of patients	Overall response rate, percentage	Complete remission, percentage
Ganem et al. (1994) [3]	27	89	37
Sawyer and Timothy (1997) [4]	11	94	38
Girinsky et al. (2001) [5]	48	81	57
Haas et al. (2003) [6]	109	92	61
Hoskin et al. (2014) [7]	243	80	48
König et al. (2018) [8]	47	90	82

The British FORT trial prospectively tested 12 × 2 Gy randomly assigned against 2 × 2 Gy in the treatment of indolent lymphomas. Owing to the superiority of the 24-Gy arm (freedom from local progression after 2 years: 93.7% versus 80.4%), recruitment was stopped before the end of the trial was reached [7]. The CR rates were 40% after LDRT (total response rate of 74%) and 60% after 24 Gy (total response rate of 81%). There were significantly more recurrences (*n* = 70) in the LDRT arm after a median follow-up time of 26 months as compared with the 24-Gy arm (21 recurrences; hazard ratio 3.42; *P* <0.0001). However, this trial has several major weaknesses (e.g., no limitation or stratification of lymphoma size; no differentiation between FL grade 1, 2, 3a, or 3b; no central pathological review; and no standardized follow-up with three-dimensional imaging) [12]. In summary, the FORT trial showed some efficacy after LDRT, but in light of the mentioned issues, it is not clear whether the difference between LDRT and 24 Gy was as large as published. In addition, no anti-CD20 antibody was applied and this might result in an increased radiosensitivity of the FL cells [13].

### Rationale for radioimmunotherapy using an anti-CD20 antibody

Several studies combined RT with systemic chemotherapy in early-stage FL. Most studies failed to demonstrate a benefit of combined therapy [13–16]. In one study, the sequential administration of COP, CHOP-B, and IF irradiation improved relapse-free but not overall survival in comparison with the historical cohort. Relapse-free survival after 10 years was 72%; however, 22% of patients experienced a grade IV neutropenia and 14% secondary malignancies were observed [17, 18].

With the development of the monoclonal chimeric anti-CD20 antibody rituximab, treatment of FL has been revolutionized in the last decade. A pivotal phase II trial tested rituximab monotherapy in 37 patients with refractory or relapsed FL. The ORR was 46% and the CR rate was 8% [19].

Also, rituximab may enhance radiosensitivity of lymphoma cells and thus may improve the efficacy of RT [20]. Additionally, rituximab maintenance has been shown to prolong progression-free survival (PFS) after first-line therapy of advanced stage FL [21] and therefore may contribute to the elimination of minimal disease that is not covered by the radiation field.

A recently published study reported a superior PFS rate with IF-RT and combined immunotherapy with R-CVP (rituximab, cyclophosphamide, vincristine sulfate, and prednisone) compared with IF-RT alone, showing that additional systemic therapy reduces out-of-field relapse and therefore might be an important component for early-stage treatment [22].

The hypothesis that rituximab in combination with IF-RT might prevent out-of-field relapses in early-stage nodal FL was investigated in a prospective, multi-center phase II study in patients with FL (World Health Organization (WHO) grades 1 and 2 in stages I and II): the MIR (MabThera^®^ and Involved field Radiation) trial. Treatment consisted of four cycles of rituximab 375 mg/m^2^ weekly up-front followed by a 4-week break with an interim staging. Patients with CR received an IF-RT of 30 Gy (3 weeks), and patients with PR or stable disease received an additional boost of 10 Gy (cumulative IF-RT dose of 40 Gy). During RT, another four weekly cycles of rituximab were applied [23]. The efficacy was comparable to that of the superior arm of the ARO98–01 trial (TLI) but with a lower morbidity profile. The PFS rate after 2 years was 85%. The CR rate after four cycles of rituximab (without radiation) was 29% in patients with macroscopic disease at inclusion. The best morphologic response was reached at month 6 with a CR rate of 79%. Response duration was associated with a continuous minimal residual disease (MRD) response, and although only few relapsing patients had follow-up samples for MRD evaluation, it seems that MRD reappearance is associated with clinical relapse [24]. The next-generation anti-CD20 antibody obinutuzumab, a fully humanized monoclonal antibody, has achieved improved response rates compared with rituximab [25, 26]. It might therefore be an ideal partner for combined radioimmunotherapy and possibly offer a better control of occult disease.

### FDG-PET/CT for staging and response evaluation

According to the results of the PRIMA trial and the FOLL05 trial, positron emission tomography/computed tomography (PET/CT) scanning possessed a high negative predictive value with a significantly superior PFS rate for PET-negative patients as compared with PET-positive patients (71% versus 33%; *P* <0.001 after 42 months, PRIMA trial [27] and 66% versus 35%; *P* <0.001 after 3 years, FOLL05 trial [28]). In regard to only morphologic changes by CT, the prognostic difference between CR and non-CR was much weaker (3-year PFS rate of 63% versus 51%; *P* = 0.04) [28]. Metabolic CR proved to be an independent prognostic marker for PFS. The prognostic value was highest in the case of a negative fludeoxyglucose-PET (FDG-PET) and a morphologic PR in the CT scan [28].

Additionally, sensitivity and specificity of an FDG-PET examination for staging purposes are very high and sensitivity was 91%–98% [29–31]. In patients in the FOLL05 trial who were staged by FDG-PET, 32% more involved lymph node regions were detected and a stage shift into a more advanced stage was diagnosed in 11% [32]. Current guidelines recommend FDG-PET for staging and response evaluation in patients with FL [33, 34], especially for clinical trials if the primary endpoint is CR.

## Methods/Design

### Study design

The study is a prospective, non-randomized, open, national, multi-center phase II trial which will be conducted at 15 locations (15 sites of radiation oncology and 15 sites of hematology and oncology).

### Objectives

The rate of metabolic CR (based on FDG-PET/CT) after low-dose involved site radiotherapy (IS-RT) with 2 × 2 Gy in combination with obinutuzumab in early-stage nodal FL will be evaluated with the aim of avoiding conventional full-dose RT. The efficacy and safety of a response-adapted radiation dose treatment schedule will be analyzed.

### Endpoints

The primary endpoint is the rate of metabolic CR (based on FDG-PET/CT) after combined radioimmunotherapy in week 18. Secondary endpoints are the morphologic CR rate in weeks 7 and 18 and month 6, PFS, toxicity, recurrence rate, recurrence patterns, overall survival, and quality of life. Additional MRD response is assessed from peripheral blood samples. Finally, a historical comparison of the early morphologic response with data of the MIR trial (using MabThera^®^/rituximab) is planned to compare efficacy of the two different antibodies.

### Additional scientific program

Genetic and molecular examinations of diagnostic lymphoma specimen will be tested for different subgroups. The detection of certain genetic profiles, which might predict response to LDRT, is one of the goals of these studies.

### Inclusion criteria

Patients (at least 18 years old, ECOG 0–2) who have centrally reviewed CD20-positive FL grade 1/2 (WHO classification) with clinical stages I or II (Ann Arbor classification) and nodal lymphoma (≤7 cm in diameter) and who have not received any prior treatment are eligible. Staging will be assessed by an initial FDG-PET/CT scan.

### Exclusion criteria

Patients with a previous extra-nodal manifestation of cancer in their medical history (exclusion: basalioma, spinalioma, melanoma *in situ*, bladder cancer T1a, or a non-metastasized solid tumor in constant remission, which was diagnosed more than 3 years ago), concomitant diseases (congenital or acquired immune-deficiency syndromes, active infections including viral hepatitis or significant cardiovascular or pulmonary disease, or severe psychiatric disease), pregnancy/lactation, or a known hypersensitivity against obinutuzumab or drugs with similar chemical structure are excluded from the trial.

### Study intervention and timeline of the study

**Fig. 1:**
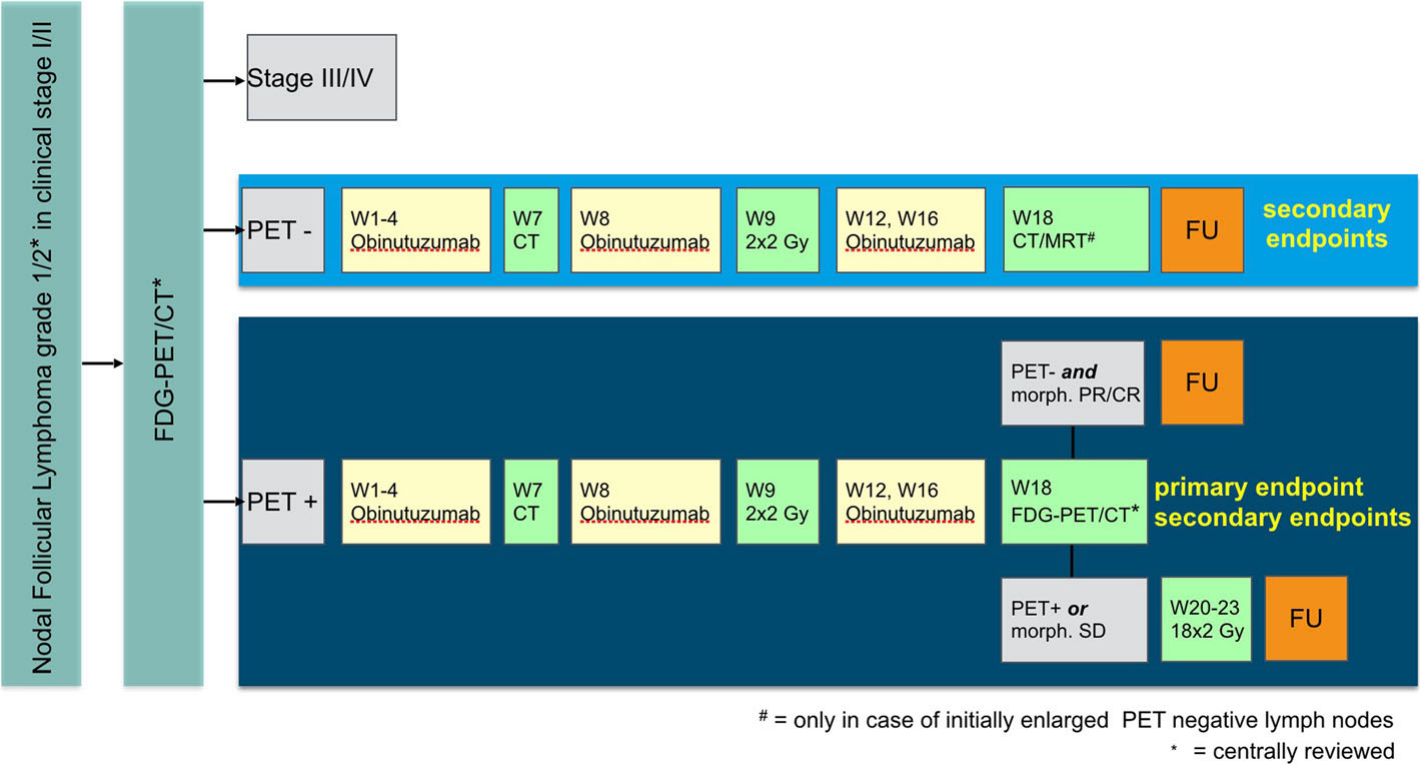
The study flow chart shows the two stage screening with 1) centrally approved histology and CT or MRI staging and 2) FDG-PET/CT as well as the further stratification and follow up

The medication obinutuzumab will be applied as a weekly 1000-mg intravenous flat dose in weeks 1–4, 8, 12, and 16. IS-RT with a dose of 2 × 2 Gy is applied to involved lymph node sites on two consecutive days (after the fifth administration of obinutuzumab) in week 9. At week 18, restaging is performed. If a sufficient response after LDRT (metabolic CR and morphologic PR/CT) is not reached, patients will receive a completion of the “full” radiation dose. This salvage RT is performed with an additional dose of 18 × 2 Gy (5 × 2 Gy per week) starting from week 20 (without obinutuzumab). The timeline is displayed in Fig. 1.

The individual follow-up for each participant is 30 months. Patients may enter in a succeeding register phase (extended follow-up) according to the standards of each participating center. The trial started in Q2/2018 and will last about 5.5 years.

### Assessment of therapeutic efficacy

Metabolic remission is assessed according to the revised (2014) criteria of Cheson et al. with the use of the 5-point score system for PET/CT uptake [33, 34], morphologic remission by CT/magnetic resonance imaging (CT/MRI) according to the National Cancer Institute (NCI) criteria [35], classification of side effects according to the NCI-Common Terminology Criteria (NCI-CTC) version 4.03, and quality of life using the EORTC QLQ-C30 (European Organization for Research and Treatment of Cancer Quality of Life Questionnaire) and the FACT-Lym (Functional Assessment of Cancer Therapy - Lymphoma) questionnaire.

### Minimal residual disease analysis

MRD will be analyzed initially, at week 18, and at months 6, 12, 18, and 24 using the markers t(14:18) PCR for MBR, 3′mbr, 5′mcr, and MCR; clonal IGH rearrangements (FR1–3); and clonal IGL rearrangements (IGK and Kappa-KDE).

### Statistical considerations

All patients who received at least parts of the treatment will be analyzed in the intention-to-treat (ITT) population and in the safety population. All patients who deviated less than 20% from the protocol-defined dose and comply with all inclusion and exclusion criteria will be analyzed in the per-protocol (PP) population. Efficacy will be evaluated in the ITT and PP populations. Toxicity data will be analyzed in the safety population.

Calculation of the number of patients to be recruited is based on the ITT population. The primary endpoint is the rate of metabolic CR in week 18 in patients with initially remaining lymphoma after the diagnostic surgery as judged by FDG-PET/CT. Given the morphologic CR rate of 37%–84% after LDRT documented in the literature and a lack of data for metabolic CR after LDRT, a CR rate of 60% is assumed. If 50 patients enter the FDG-PET/CT and the observed metabolic CR rate amounts to 60%, the half width of the asymptotic two-sided 95% confidence interval amounts to about ±13.5%. Based on the experience of the MIR trial, a general dropout rate of 10% is assumed, and about 30% of the included patients will not have remaining lymphoma after initial surgery. In addition, owing to stage shifting to stage III/IV disease, a dropout rate of about 15% after the initial FDG-PET/CT is expected.

These considerations lead to the calculation that a maximum of 93 patients have to be included in the trial so that at least 50 patients will be available for final assessment of the primary endpoint. The recruitment will be stopped if 79 patients have a FDG-PET/CT confirmed stage I/II FL and a trial-specific therapy has been initiated or 55 patients had a second restaging FDG-PET/CT in week 18 or 93 patients were included (whatever occurs first). Trial data are evaluated by applying methods of descriptive data analysis.

### Ethics

The procedures set out in this trial protocol are designed to ensure that all persons involved in the trial abide by the International Conference on Harmonization (ICH) harmonized tripartite guideline on Good Clinical Practice (ICH-GCP) and the ethical principles described in the applicable version of the Declaration of Helsinki. The trial will be carried out in keeping with local legal and regulatory requirements. The regulations of the AMG and GCP regulations and the Bundesdatenschutzgesetz (BDSG) will be respected. Before the start of the trial, all documents were submitted to the independent ethics committee and the competent authority (PEI). The local ethics committee approved this study on June 6, 2016 (AFmu-316/2017).

### Data quality assurance

The histologic diagnosis must be centrally verified by one of the reference pathologists of the German Lymphoma Alliance and the inclusion PET/CT scan by an experienced radiologist (CT/MRI) and specialist for nuclear medicine (PET).

## Discussion

The MIR trial has shown that, in early-stage FL treated by immunoradiotherapy, the radiation volumes could be significantly reduced without compromising effectiveness as compared with historical large field irradiation. Also, the toxicity of the combined approach of an anti-CD20 antibody and IF-RT was much lower than the historical data.

**Fig. 2:**
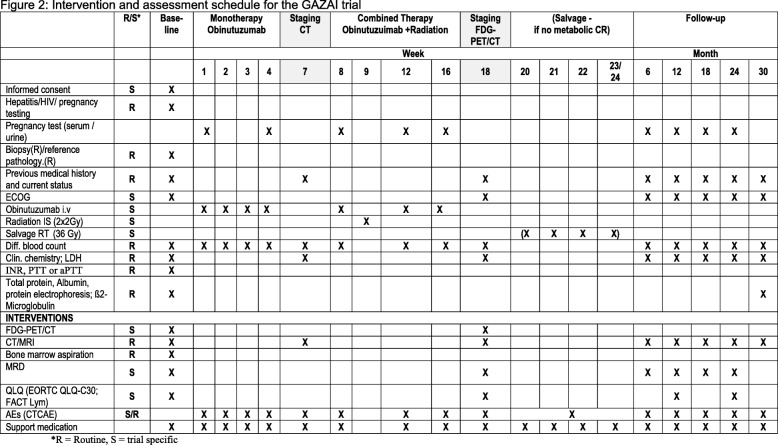
Intervention and assessment schedule for the GAZAI (GAZyvaro and response Adapted Involved-site Radiotherapy) trial. *Abbreviations*: *AE* adverse event, *aPTT* activated partial thromboplastin time, *CR* complete response, *CT* computed tomography, *CTCAE* Common Terminology Criteria for Adverse Events, *ECOG* Eastern Cooperative Oncology Group, *EORTC* European Organization for Research and Treatment of Cancer, *FACT Lym* Functional Assessment of Cancer Therapy - Lymphoma, *FDG-PET/CT* fludeoxyglucose positron emission tomography/computed tomography, *INR* international normalized ratio, *IS* involved site, *LDH* lactate dehydrogenase, *MRD* minimal residual disease, *MRI* magnetic resonance imaging, *PTT* partial thromboplastin time, *QLQ* Quality of Life Questionnaire, *RT* radiotherapy

The GAZAI (GAZyvaro and response Adapted Involved-site Radiotherapy) trial (Fig. 2) is designed to investigate whether a response-adapted reduction of the radiation dose to 2 × 2 Gy in combination with the anti-CD20 antibody obinutuzumab will yield similar response rates and to prospectively explore the concept of immunomodulatory LDRT in nodal FL. Additional information can be found online at the SPIRIT 2013 checklist see Additional file 1.

## Trial status

Protocol version 1.2a (01.05.2018). The trial started in April 2018 and is currently recruiting. The length of clinical phase is about 66 months. (The planned end of the study is the end of 2023.)

## Additional file

Additional file 1: SPIRIT (Standard Protocol Items: Recommendations for Interventional Trials) 2013 Checklist: Recommended items to address in a clinical trial protocol and related documents. (DOC 236 kb)

## Data Availability

The data used in this analysis are from publications available in the public domain.

## Abbreviations

CR: Complete response
CT: Computed tomography
FDG: Fludeoxyglucose
FL: Follicular lymphoma
GCP: Good Clinical Practice
ICH: International Conference on Harmonization
IF: Involved field
IS: Involved site
ITT: Intention-to-treat
LDRT: Low-dose radiotherapy
MIR: MabThera^®^ and Involved field Radiation
MRD: Minimal residual disease
ORR: Overall response rate
PET: Positron emission tomography
PFS: Progression-free survival
PP: Per-protocol
PR: Partial response
RT: Radiotherapy/Radiation therapy
WHO: World Health Organization
