# Supplementary material for: Therapy of nodal Follicular Lymphoma (WHO grade 1/2) in clinical stage I/II using response adapted Involved Site Radiotherapy in combination with Obinutuzumab (Gazyvaro) - GAZAI Trial (GAZyvaro and response adapted Involved-site Radiotherapy): a study protocol for a single-arm, non-randomized, open, national, multi-center phase II trial
Source: Trials. 2019 Aug 30;20:544. doi: 10.1186/s13063-019-3614-y (PMC6717383; doi:10.1186/s13063-019-3614-y)
Supplement: Supplementary file 1 — SPIRIT (Standard Protocol Items: Recommendations for Interventional Trials) 2013 Checklist: Recommended items to address in a clinical trial protocol and related documents. (DOC 236 kb) [file 13063_2019_3614_MOESM1_ESM.doc]

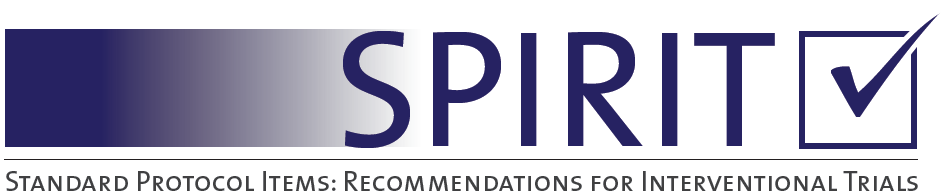


SPIRIT 2013 Checklist: Recommended items to address in a clinical trial protocol and related documents*

| Section/item | Item No | Description | Addressed on page number |
| --- | --- | --- | --- |
| **Administrative information** | | |  |
| Title | 1 | Therapy of Nodal Follicular Lymphoma (WHO grade 1/2) in Clinical Stage I/II using Response Adapted Involved Site Radiotherapy in Combination with Gazyvaro - GAZAI Trial (GAZyvaro and response Adapted Involved-site Radiotherapy) | 1 |
| Trial registration | 2a | GAZAI Trial  EudraCT: 2016-002059-89  ClinicalTrials (NCT): NCT03341520 | 1, 4-5 |
|  | 2b | N/A |  |
| Protocol version | 3 | 1.2a (01.05.2018) | 12 |
| Funding | 4 | The trial is partially supported by Roche Pharma AG, Switzerland. Support includes trial organization and monitoring by the KKS Heidelberg, the statistical analysis, data management and the supply of the study medication. There is no other funding of the trial. The authors have no financial relationship with Roche Pharma AG. The authors also participate in other scientific trials which are supported by Roche Pharma AG. | 13 |
| Roles and responsibilities | 5a | **Authors:**  Laila König^1,2^, Martin Dreyling^3^, Jan Dürig^4^, Marianne Engelhard^5^, Karin Hohloch^6,7^, Andreas Viardot^8^, Mathias Witzens-Harig^9^, Meinhard Kieser^10^, Wolfram Klapper^11^, Christiane Pott^12^, Klaus Herfarth^1,2^  **Affiliations:**  ^1^Department of Radiation Oncology, University Hospital Heidelberg, Im Neuenheimer Feld 400, 69120 Heidelberg, Germany  ^2^Heidelberg Institute of Radiation Oncology (HIRO), Heidelberg, Germany  ^3^Department of Medicine III, University Hospital, Ludwig-Maximilians-University  Munich, Germany  ^4^Department of Hematology, University of Essen, Essen, Germany  ^5^Department of Radiotherapy, University Hospital of Essen, Essen, Germany  ^6^Department of Hematology and Oncology, Kantonspital Graubünden, CH-7000 Chur  ^7^Department of Hematology and Oncology, Georg August University, Göttingen, Germany  ^8^Department of Internal Medicine III, University Hospital Ulm, Ulm, Germany  ^9^Department of Hematology, Oncology and Rheumatology, University Hospital Heidelberg, Heidelberg, Germany  ^10^Institute of Medical Biometry and Informatics, University of Heidelberg, Heidelberg, Germany  ^11^Department of Pathology, Hematopathology Section and Lymph Node Registry, University of Kiel and University Medical Center Schleswig-Holstein, Kiel, Germany  ^12^Department of Medicine 2, University Hospital Schleswig-Holstein, Kiel, Germany | 1-3 |
|  | 5b | Ruprecht-Karls-Universität Heidelberg Medical Faculty represented by University Hospital Heidelberg and its Commercial Director Dipl. Volkswirtin Irmtraut Gürkan  Im Neuenheimer Feld 672, 69120 Heidelberg | n.a. |
|  | 5c | The funding source and sponsor have no role in study design, data collection, data analysis, data interpretation, or writing of the report. | n.a. |
|  | 5d | KH, MD, JD, ME, AV and MWH developed and planned this trial and KH is the principle investigator of the study. LK performed basic research and wrote the manuscript. CP is conducting for MRD analysis. WK is head of the pathological reference panel and coordinates an additional scientific program. MK is the trial statisticians and responsible for statistical planning and statistical analysis. All authors read and approved the final manuscript. | 13 |
| Introduction |  |  |  |
| Background and rationale  Objectives | 6a, 6b,  7 | s. manuscript pages 3-5 | 3-5 |
| Trial design | 8 | The study is a prospective, non-randomized, open, national multicenter phase II trial, which will be conducted at 15 locations (15 sites of Radiation Oncology and 15 sites of Hematology&Oncology). | 8 |
| Methods: Participants, interventions, and outcomes | | |  |
| Study setting | 9 | s. manuscript pages 6-8  Access and availability of services are equal between the different sites. Procedures (e.g. drug applications, radiation therapy) have been defined as standard procedures as explained in the study protocol. All sites have been trained before start of the trial and are supervised in periodic intervals. | 6-8 |
| Eligibility criteria | 10 | Inclusion Criteria  • Centrally reviewed CD20-positive follicular lymphoma grade 1/2 based on WHO classification (2008)  • Untreated (radiation-, chemo- or immunotherapy) nodal lymphoma  • Age: ≥18 years  • ECOG: 0-2  • Stage: clinical stage I or II (Ann Arbor classification)  • Risk profile: Largest diameter of the lymphoma < 7 cm (sectional images)  • Written informed consent and willingness to cooperate during the course of the trial  • Adequate hematologic function (unless abnormalities are related to NHL)  • Capability to understand the intention and the consequences of the clinical trial  • Adequate contraception for men and women of child-bearing age during therapy and 18 months thereafter  • Patients with non-active hepatitis B infection (HBsAg neg/HBcAB pos/HBV DNA neg) under 1- year prophylactic anti-viral therapy (e.g. Entecavir®) possible (see also 5.6. Prior and Concomitant Disease) Exclusion Criteria  • Extra nodal manifestation  • Secondary cancer in the patient’s medical history  • Concomitant diseases: congenital or acquired immune-deficiency syndromes, active infections including viral hepatitis, uncontrolled concomitant diseases including significant cardiovascular or pulmonary disease  • Severe psychiatric disease  • Pregnancy / lactation  • Known hypersensitivity against Gazyvaro (Obinutuzumab) or drugs with similar chemical structure or any other additive of the pharmaceutical formula of the study drug  • Participation in another interventional trial or follow-up period of a competing trial which can influence the results of this current trial  • Creatinine > 1.5 times the upper limit of normal (ULN) or calculated creatinine clearance < 40 mL/min, AST or ALT > 2.5 × ULN, Total bilirubin ≥ 1.5 × ULN, INR > 1.5 × ULN, PTT or aPTT > 1.5 × the ULN | 9-10 |
| Interventions | 11a | The medication Gazyvaro (Obinutuzumab) will be applied in a dose of 1000mg i.v. flat dose weekly in weeks 1-4 and week 8, week 12, week 16.  IS-RT with a dose of 2 x 2 Gy is applied to involved lymph node sites on two consecutive days (after 5th administration of Gazyvaro) in week 9. At week 18 restaging is performed. If a sufficient response after LDRT (metabolic CR and morphologic PR/CT) is not reached, patients will receive a completion of the “full” radiation dose. This salvage radiotherapy is performed with an additional dose of 18 x 2 Gy (5 x 2 Gy/week) starting from week 20 (without Gazyvaro). The timeline is displayed in figure 1 (see below)  The individual follow up for each participant is 30 months. Patients may enter in a succeeding register phase (extended follow up) according to the standards of each participating center. The trial started in Q2/2018 and will last approximately 5.5 years.  See figure 1 below | 10 |
|  |  |  |  |
|  | 11b | Management of IRRs may require temporary interruption, reduction in the rate of infusion, or treatment discontinuations of Gazyvaro as outlined below.  • Grade 4 (life threatening): Infusion must be stopped and therapy must be permanently discontinued.  • Grade 3 (severe): Infusion must be temporarily stopped and symptoms treated. Upon resolution of symptoms, the infusion can be restarted at no more than half the previous rate (the rate being used at the time that the IRR occurred) and, if the patient does not experience any IRR symptoms, the infusion rate escalation can resume at the increments and intervals as appropriate for the treatment dose (see above). The infusion must be stopped and therapy permanently discontinued if the patient experiences a second occurrence of a Grade 3 IRR.  • Grade 1-2 (mild to moderate): The infusion rate must be reduced and symptoms treated. Infusion can be continued upon resolution of symptoms and, if the patient does not experience any IRR symptoms, the infusion rate escalation can resume at the increments and intervals as appropriate for the treatment dose  Patients must not receive further Gazyvaro infusions if they experience:  • acute life-threatening respiratory symptoms,  • a Grade 4 (i.e. life threatening) IRR or,  • a second occurrence of a Grade 3 (prolonged/recurrent) IRR (after resuming the first infusion or during a subsequent infusion). Patients who have pre-existing cardiac or pulmonary conditions should be monitored carefully throughout the infusion and the post-infusion period. Hypotension may occur during Gazyvaro intravenous infusions. Therefore, withholding of antihypertensive treatments should be considered for 12 hours prior to and throughout each Gazyvaro infusion and for the first hour after administration. Patients at acute risk of hypertensive crisis should be evaluated for the benefits and risks of withholding their anti-hypertensive medicine. | n.a. |
|  | 11c | Roche Pharma AG will provide the quantity of trial medication required for the clinical trial. The medication provided must be used only in the context of this clinical trial. Careful records will be kept of the trial medication supplied to the centers and distributed to the patients. At the end of the study, all unused medication will be returned to manufacturer. If deficiencies of the trial medication are noticed, the monitor, the project manager and the LKP must be informed immediately. The order and delivery of the mediation includes the local pharmacy of each site. The order fax for the first 4 applications of each patient will be sent by the trial center in Heidelberg, the order form for week 8, 12, and 16 by the treating site Monitoring will be done by on-site visits by a clinical monitor according to SOPs of the KKS. The monitor will review the entries into the CRFs on the basis of source documents. The investigator must allow the monitor to verify all essential documents and must provide support at all times to the monitor. The monitor will document the visit in a report for the sponsor. The site will be provided with a follow-up letter of the findings and the necessary actions to be taken. By frequent communications (letters, telephone, fax, e-mail), the site monitor will ensure that the trial is conducted according to the protocol and regulatory requirements. Frequency and details of monitoring will be defined in the monitoring manual. If there are major findings during monitoring or an audit, the investigational site might be closed by the LKP. | n.a. |
|  | 11d | also see 10.  Relevant additional diseases present at the time of informed consent are regarded as concomitant diseases and will be documented on the appropriate pages of the case report form (CRF). Included are conditions that are seasonal, cyclic, or intermittent (e.g. seasonal allergies; intermittent headache). Abnormalities, which appear for the first time or worsen (intensity, frequency) during the trial are adverse events (AEs) and must be documented on the appropriate pages of the CRF. Prior and Concomitant Medication The treatment of accompanying illnesses not subject to the exclusion criteria is permissible if this is not expected to have any effect on the outcome measures used in this study and to interfere with the trial medication. If concomitant drugs are administered, these must be recorded in the patient file and in the CRF. All patients must get a Hepatitis B testing before treatment with Gazyvaro. At least the HBsAg status and the HBcAb status should be assessed and can be added by other markers according to the local recommendations. In case of HBsAg negative/HBcAb positive status, also serology for HBV DNA has to be done. Patients suffering from an active Hepatitis B infection (all HBsAg positive and all HBcAb positive/HBV-DNA positive) must not be treated with Gazyvaro and have to be excluded. Patients with HBcAb positive / HBV DNA negative status should be referred to a hepatologist or gastroenterologist before start of treatment and should be monitored and managed following local standards to prevent hepatitis reactivation. It is recommended to do a 1-year prophylaxis with antiretroviral medication (e.g. Entecavir®). If there is no chance of prophylaxis, the patient must be excluded. | n.a. |
| Outcomes | 12 | Primary endpoint is the metabolic complete response (CR) in week 18 in patients with initially remaining lymphoma judged by FDG-PET/CT.  Secondary endpoints are:  • Morphologic CR, PR, SD, PD in week 7, week 18 and month 6 in patients with initially remaining lymphoma judged by CT/MRI  • Historical comparison of the morphologic response with MIR data (using MabThera); the comparison of the CR rate in week 7 would be a comparison of the two different CD20 antibodies.  • Progression-free survival (PFS) of all treated patients (2 years after individual treatment start)  • Toxicity (NCI-CTC criteria, version 4.03) of all patients  • Relapse rate and pattern of recurrence of all treated patients at all follow-up visits.  • Overall survival (OS) of all treated patients (2 years)  • Quality of life according EORTC QLQ C30 and FACT-Lym questionnaires at inclusion and in week 18, month 12 and 24 (all treated patients)  • MRD response: initially, week 18, months 6, 12, 18 and month 24 (all treated patients). MRD is evaluated using at least the markers: t(14:18) PCR for MBR, 3’mbr, 5’mcr and MCR; clonal IGH rearrangements (FR1-3); clonal IGL rearrangements (IGK and Kappa-KDE) | 9 |
| Participant timeline | 13 | s. fig. 1  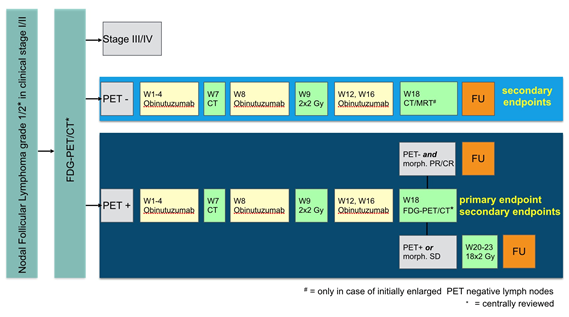 | fig. 1 |
| Sample size | 14 | All patients, who received at least parts of the treatment, will be analyzed in the intention-to-treat (ITT) population as well as in the safety population. All patients, who deviated less than 20% from the protocol-defined dose and fulfill all inclusion and exclusion criteria, will be analyzed in the per-protocol (PP) population. Efficacy will be evaluated in the ITT as well as in the PP population. Toxicity data will be analyzed in the safety population.  Calculation of the number of patients to be recruited is based on the ITT population. Primary endpoint is the rate of metabolic CR in week 18 in patients with initially remaining lymphoma after the diagnostic surgery as judged by FDG-PET/CT. Based on the morphologic CR rate of 37-84% after LDRT documented in the literature and in face of a lack of data for metabolic CR after LDRT, a CR rate of 60% is assumed. If 50 patients enter the FDG-PET/CT and the observed metabolic CR rate amounts to 60%, the half width of the asymptotic two-sided 95% confidence interval amounts to about ± 13.5%. Based on the experience of the MIR trial, a general drop-out rate of 10% is assumed, and about 30% of the included patients will not have remaining lymphoma after initial surgery. In addition, a drop-out rate of about 15% after the initial FDG-PET/CT due to stage-shifting to stage III/IV disease is expected.  These considerations lead to the calculation that a maximum of 93 patients have to be included in the trial so that at least 50 patients will be available for final assessment of the primary endpoint. The recruitment will be stopped, if (whatever occurs first): 79 patients will have a FDG-PET/CT confirmed stage I/II follicular lymphoma and a trial specific therapy has been initiated or 55 patients had a second restaging FDG-PET/CT in week 18 or 93 patients were included. Trial data are evaluated by applying methods of descriptive data analysis. | 10-11 |
| Recruitment | 15 | high volume medical centres, 15 centres | 9 |
| **Methods: Assignment of interventions (for controlled trials)** | | |  |
| Allocation: |  |  |  |
| Sequence generation | 16a | n.a., no randomization | n.a. |
| Allocation concealment mechanism | 16b | n.a., no randomization | n.a. |
| Implementation | 16c | n.a., no randomization | n.a. |
| Blinding (masking) | 17a | n.a., no blinded treatment | n.a. |
|  | 17b | n.a., no blinded treatment | n.a. |
| **Methods: Data collection, management, and analysis** | | |  |
| Data collection methods,  Data management | 18a, 18b and 19 | All entries in the CRF will be verifiable by source documents. In advance, exceptions to this rule can be defined by the sponsor. A detailed list will be provided in the Investigator Site File. Regardless, there must be a minimum documentation, which provides information on study participation and includes all medical information necessary for appropriate medical care outside of the clinical trial in the patient record. In addition, source documents must mention that the patient has been included in an investigational study. Finally, there must be no data that are inconsistent between CRF and source documents. The investigator is responsible for ensuring that all sections of the CRF are completed correctly and that entries can be verified against source data. Any errors should have a single line drawn through them so that the original entry remains legible and the correct data should be entered at the side with the investigator's signature, date and reason for change. Self-explanatory corrections need not to be justified. The correctness of entries in CRF will be confirmed by dated signature of the responsible investigator. The original CRF will be transferred to the data management in the Institute of Medical Biometry and Informatics (IMBI) Heidelberg; one copy will be remained by the investigator.  Data Handling  All data will be entered in a database as recorded in the CRF by trained staff of the IMBI. To ensure data quality, a double data entry will be done. The data management will check completeness, validity, and plausibility of data by validation programs, which will generate queries. All missing data or inconsistencies will be reported back to the centre(s) and have to be clarified by the responsible investigator prior to database lock. If no further corrections are to be made in the database. it will be declared locked and used for statistical analysis. All data management activities will be done according to the current SOPs of the IMBI. Original files of the CRF will be transferred to the LKP after analysis and completion of the trial report. |  |
| Statistical methods | 20a-c | Statistical methods for analysing primary and secondary outcomes. Reference to where other details of the statistical analysis plan can be found, if not in the protocol. There will be a descriptive analysis of the data. The rate of metabolic CR in week 18 will be calculated as relative frequency. The related 95% confidence interval will be calculated using the “score method” by Wilson. This method showed excellent performance characteristics as compared to alternative approaches of constructing confidence intervals for rates. In addition, the rate of metabolic CR will be estimated using the Kaplan-Meier method and the 95% Greenwood confidence interval to take into account a potential influence of censored values. (ITT and PP population) PFS and OS at 2 years after initiation of the therapy of each patient will be analyzed using the Kaplan-Meier method and the two-sided 95% confidence intervals according to Greenwood. (ITT and PP population). Remission- and recurrence rates will be calculated as relative frequencies and reported together with the 95% confidence intervals according to Wilson. (ITT and PP population) Quality of life will be evaluated according to the evaluation guide of the two questionnaires. (ITT and PP population). Toxicity will be evaluated for frequency and intensity (safety population). Adverse events will be coded by MedDRA (actual version at the time of data base lock). The counts of the events and the counts of patients will be listed in a table. All patients under observation will be taken into account for the calculations of the frequency of recurrences at the respective time. All statistical procedures will be defined in a statistical analysis plan (SAP), which will be finalized before data base lock. The responsible statistician and the LKP will authorize the SAP. | 10-11 |
| **Methods: Monitoring** | | |  |
| Data monitoring  Harms,  Auditing | 21a-b,  22,  23 | The DMC consists of independent experts in the treatment of malignant lymphomas. The DMC should evaluate the conduct of the trial, the safety, and the achievements of the major endpoints. The duty of the DMC is to secure the ethically correct conduct of the trial and to protect the interests of safety for the patients. The DMC will meet on a regular basis (GLSG annual meetings). The DMC will give recommendations to the LKP regarding modifications, stopping or continuation of the clinical trial. Those recommendations should be send to the LKP in written form within 2 months after the meeting. The DMC will get the annual DSURs. The members of the DMC are listed in the protocol. No interim analysis is planned. The trial can be stopped by the LKP if the DMC detects an unexpected accumulation of side effects (each grade 5, 2 succeeding grade 4 or 3 succeeding grade 3 CTC side effects), has new information about the effect-risk ratio of the investigated therapy or the used methods of examination. The trial can also be stopped by the LKP if recruitment is below the expectations and cannot be improved. All investigators have to be informed immediately about a stopping or a permanent ending of the trial. The participating sites must accept the decision. A participating site can also be closed early by the LKP if the site does not act according to ICH-GCP or according to the trial protocol or the recruitment or the quality of the data is below the expectations. If the DMC suggests an interruption or an ending of the trial due to SAE evaluations or other reasons, the ethical board and the authorities (BOB and sate authority) have to be informed. The Ethical Board and the authorities can also decide about an interruption or an ending of the trial. Monitoring will be done by on-site visits by a clinical monitor according to SOPs of the KKS. The monitor will review the entries into the CRFs on the basis of source documents. The investigator must allow the monitor to verify all essential documents and must provide support at all times to the monitor. The monitor will document the visit in a report for the sponsor. The site will be provided with a follow-up letter of the findings and the necessary actions to be taken. By frequent communications (letters, telephone, fax, e-mail), the site monitor will ensure that the trial is conducted according to the protocol and regulatory requirements. Frequency and details of monitoring will be defined in the monitoring manual. If there are major findings during monitoring or an audit, the investigational site might be closed by the LKP | 10-11 |
| Ethics and dissemination | | |  |
| Research ethics approval | 24 | Plans for seeking research ethics committee/institutional review board (REC/IRB) approval  The procedures set out in this trial protocol, pertaining to the conduct, evaluation, and documentation of this trial, are designed to ensure that all persons involved in the trial abide by ICH harmonised tripartite guideline on Good Clinical Practice (ICH-GCP) and the ethical principles described in the applicable version of the Declaration of Helsinki. The trial will be carried out in keeping with local legal and regulatory requirements. The regulations of the AMG and GCP regulations as well as the EU Datenschutz-Grundverordnung (DSGVO) will be respected. The procedures set out in this trial protocol, pertaining to the conduct, evaluation, and documentation of this trial, are designed to ensure that all persons involved in the trial abide by ICH harmonised tripartite guideline on Good Clinical Practice (ICH-GCP) and the ethical principles described in the applicable version of the Declaration of Helsinki. The trial will be carried out in keeping with local legal and regulatory requirements. Before the start of the trial, the trial protocol, informed consent document, and any other appropriate documents have been submitted to the independent Ethics Committee (EC) as well as to the competent authority (PEI). A written favourable vote of the EC and an (implicit) approval by the competent authority was a prerequisite for initiation of this clinical trial. The Federal Office for Radiation Protection (Bundesamt für Strahlenschutz) has approved the investigational use of FDG-PET/CT in this trial before initiation of the trial. The use of radiation therapy in this trial has been reviewed by an radiation safety committee of experts of the DEGRO. | 13 |
| Protocol amendments | 25 | The Coordinating Investigator and all investigators will be given an up-to-date investigator’s brochure containing full details of the status of the pre-clinical and clinical knowledge of the study medication. As soon as new information is obtained, an updated version will be supplied or an amendment added to the existing investigator’s brochure. The investigator ensures that all team members are informed adequately about the protocol, all amendments to the protocol, the study procedures und study specific duties and tasks. The investigator will maintain a list to delegate tasks to the team members. | n.a. |
| Consent or assent | 26a | Local physician | n.a. |
|  | 26b | n.a. | n.a. |
| Confidentiality | 27 | The data obtained in the course of the trial will be treated pursuant to the Federal Data Protection Law (Bundesdatenschutzgesetz, BDSG). During the clinical trial, subjects will be identified solely by means of their individual identification code. Trial data stored on a computer will be stored in accordance with local data protection law and will be handled in strictest confidence. Distribution of these data to unauthorised persons has to be prevented strictly. The appropriate regulations of local data legislation will be fulfilled in its entirety. The subject consents in writing to release the investigator from his/her professional discretion in so far as to allow inspection of original data for monitoring purposes by health authorities and authorised persons (inspectors, clinical monitors, auditors). Authorised persons (inspectors, clinical monitors, auditors) may inspect the subject-related data collected during the trial ensuring the data protection law. The investigator will maintain a subject identification list (subject numbers with the corresponding subject names) to enable records to be identified. Subjects who did not consent to circulate their pseudonymised data will not be included into the trial. | 11 |
| Declaration of interests | 28 | The trial is partially supported by Roche Pharma AG, Switzerland. Support includes trial organization and monitoring by the KKS Heidelberg, the statistical analysis, data management and the supply of the study medication. There is no other funding of the trial. The authors have no financial relationship with Roche Pharma AG. The authors also participate in other scientific trials which are supported by Roche Pharma AG. | 13 |
| Access to data | 29 | According to ICH-GCP the investigator(s)/institution(s) must provide direct access to source data/documents for trial related monitoring, audits and regulatory inspection. Each subject has consented - via written informed consent - to direct access to his/her original medical records for trialrelated monitoring, audit and regulatory inspection. Any data to be recorded directly on the CRFs (i.e., no prior written or electronic record of data), and to be considered to be source data must be clearly identified (see 11.1). In the absence of either an audit-trail or limited access for the monitor the electronic record of data must be printed out (certified copy). In case of changes a new print-out has to be made. Regulatory authorities and/ or auditors authorised by the sponsor may request access to all source documents, CRFs, and other trial documentation. The investigator who must provide support at all times for these activities must guarantee direct access to these documents. The investigator will inform the sponsor immediately about a planned inspection | 11,13 |
| Ancillary and post-trial care | 30 | n.a. | n.a. |
| Dissemination policy | 31a | n.a. | n.a. |
|  | 31b | Publication will be prepared under the lead of the principal investigator of the study. The first and last authorship are reserved for the principal investigator and the study coordinator of the study if both do not wish to transfer their authorship to a third person. All data will be published independently of the results of the trial. | n.a. |
|  | 31c | n.a. | n.a. |
| Appendices |  |  |  |
| Informed consent materials | 32 | Before being admitted to the clinical trial, the patient must consent to participate after being fully informed by the investigator or a designated member of the investigating team about the nature, importance, risks and individual consequences of the clinical trial and their right, to terminate the participation at any time. The patient should also have the opportunity to consult the investigator, or a physician member of the investigating team about the details of the clinical trial. The informed consent to participate in the clinical trial may be withdrawn by the patient verbally in the presence of, or in written form directed to, the investigator or a physician member of the investigating team at any time during the trial. The patient must not entail any disadvantage therefore or be coerced or unduly influenced to continue to participate. Furthermore, the patient is not obligated to disclose reasons for the withdrawal of the consent. If the patient has a primary physician, the investigator should inform him or her about the patient’s participation in the trial, provided the patient agrees hereto. After reading the informed consent document, the patient must give consent in writing. The patient's consent must be confirmed by the personally dated signature of the patient and by the personally dated signature of the physician conducting the informed consent discussion. If the patient is unable to write, oral presentation and explanation of the content of the informed consent form and of the data protection information must take place in the presence of an impartial witness. The witness and the physician conducting the informed consent discussions must also sign and personally date the consent document. The witness must not be in any way dependent on the sponsor of the trial, the trial site or any member of the investigating team (e. g. an employee at the trial site.). A copy of the signed informed consent document must be given to the subject; the original will be filed by the investigator. The documents must be in a language understandable to the subject and must specify who informed the subject. The subjects will be informed as soon as possible if new information may influence his/her decision to participate in the trial. The communication of this information should be documented. The informed consent for genetic profiling and the extended follow-up according to a patient register will be independent of the consent of participation in the trial | 13 |
| Biological specimens | 33 | MRD (minimal residual disease) analysis: 10 ml EDTA-blood and 10 ml STRECK tube for cell-free DNA collection before the first administration of Gazyvaro, Week 18, Month 6 /12 / 18 / 24  All samples will be pseudonymized using the patient ID code and send to the analysing central laboratory in Genetic and molecular investigations in relation to lymphoma disease are planned on the initial biopsy specimen as additional scientific program. These investigations are planned and coordinated by the pathology panel of the GLSG. All investigations should serve the increase of knowledge about origin, expansion and therapy of malignant lymphomas. There might be new scientific questions/methods concerning the aim of the study, which cannot be addressed today (e.g. knowledge of a new genetic mutation in follicular lymphomas, new measurement tools, which allow a more sensitive evaluation of MRD). | 10 |

*It is strongly recommended that this checklist be read in conjunction with the SPIRIT 2013 Explanation & Elaboration for important clarification on the items. Amendments to the protocol should be tracked and dated. The SPIRIT checklist is copyrighted by the SPIRIT Group under the Creative Commons “[Attribution-NonCommercial-NoDerivs 3.0 Unported](http://www.creativecommons.org/licenses/by-nc-nd/3.0/)” license.
